# Recent advances in pediatric uroradiology

**DOI:** 10.4103/0970-1591.36713

**Published:** 2007

**Authors:** Pramod P. Reddy

**Affiliations:** Division of Pediatric Urology, Surgical Services, Cincinnati Children's Hospital Medical Center, Cincinnati, Ohio 45229, USA

**Keywords:** Imaging, pediatric, uroradiology

## Abstract

Pediatric Urology is a surgical subspecialty that is very dependent upon radiographic imaging as the majority of the genitourinary (GU) tract is internally located. Technological advances in various imaging modalities (e.g. ultrasonography, nuclear medicine, CT and MRI) have aided in our ability to visualize and evaluate the functionality of the GU tract, enabling the diagnosis of various disease processes that affect the genitourinary system. Collectively the advances in uro-radiology have improved our understanding of the natural history of many conditions that involve the GU tract. As a result of these newer imaging modalities, some of the more traditional techniques have assumed a limited role in the diagnostic evaluation of the pediatric GU patient (e.g. intravenous urography).

The purpose of this article is to review the advances in radiographic imaging, in particular the cross-sectional imaging modalities and discuss their utility (appropriate indications and application) in Pediatric Urology, so that the reader can maximize the diagnostic yield of these studies. For a thorough review of any of the imaging modalities discussed in this article and their utility in the practice of pediatric urology, I would direct the readers to articles in the radiological literature that are specific to that technology. Besides the obvious technological advances in imaging modalities, this review also discusses the attention to radiation safety for the pediatric patient that every physician who orders a diagnostic imaging study in a child should be aware of.

The field of radiology has evolved immensely over the course of the last 112 years since Wilhelm Röntgen took the first radiographic image of his wife's hand. The exploding field of diagnostic imaging is one of the most rapid and remarkable examples of translational research; bench to bedside research. There are now multiple modalities available to image the human body in great detail, which we as surgeons can use to improve the outcomes for the patients that we have the privilege of treating. The indications for diagnostic imaging usually depend upon the clinical presentation and the age of the patient. Frequently more than one imaging technique is required to fully evaluate the anatomy and physiology of the genitourinary (GU) tract. At the end of this article I will provide a pictorial review of the imaging modalities discussed along with the clinical history of the patients whose radiographic results are presented.

The various imaging modalities utilized in the field of Pediatric Urology include:

- Plain radiography
- Contrast studies (with fluoroscopy)
- Ultrasound (US)
- Computerized tomography (CT scans)
- Nuclear medicine imaging
- Magnetic resonance imaging (MRI)
- Rotational fluoroscopic tomography (RFT)
- Positron emitted tomography (PET scan)

## PLAIN RADIOGRAPHY

Plain radiographs utilize the difference in radiographic density of the various body parts to create an image or a radiograph. Traditionally these images were captured on X-ray film, however, nowadays, most institutions have implemented film-less digital imaging systems. The advantages of this shift in technology are the improved resolution of the images and the ability to instantly and remotely share access to these images using a picture archiving and communication system (PACS). One of the main advances of this imaging modality has been an increased awareness of the danger of cumulative radiation exposure in the pediatric patient (especially in children with chronic health conditions that require long-term follow-up and repeated imaging). The risk of developing a lethal cancer from radiation exposure in children is theorized to be two to four times higher than for adults per dose unit.[1,2] While the exact reasons for the significant differences in the risk of developing neoplasms based on age are unclear, it is thought to be due to a combination of the following facts:

- Children have greater cell proliferation rates, especially during physiological growth spurts and are therefore much more radiosensitive than adults.
- Children have longer life expectancy from the time of the radiation exposure, allowing for radiation-induced chromosomal mutations to become clinically relevant.
- For any given radiographic procedure, the effective radiation dose is larger in a small infant than in an adult.

In the USA. numerous national societies have mandated new guidelines for the dosimetry in pediatric patients, with the introduction of the ‘ALARA’ principle, which mandates that doses should be As Low As Reasonably Achievable.[3] The reduced dosimetry balances the risk of radiation exposure to the patient with the need for adequate imaging quality. Finally, I should state that in cases where diagnostic study is positively indicated, the risk of the procedure is far outweighed by the potential health benefit of appropriate diagnosis and treatment and no child should be denied appropriate diagnostic evaluation.

**Figure 1:**
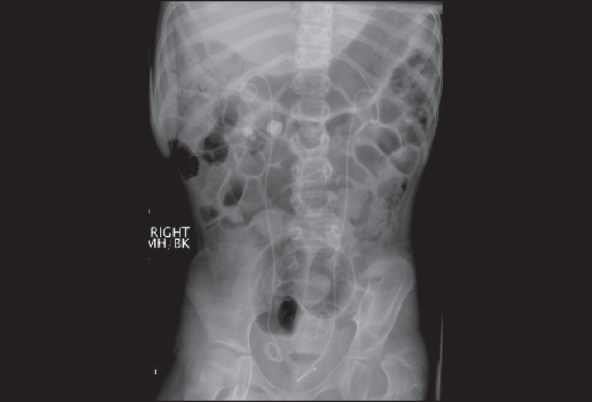
Plain film KUB. Male child with profound developmental disabilities. Patient presented with bilateral obstructing renal calculi. Patient underwent placement of bilateral ureteral stents and staged extracorporeal shockwave lithotripsy. KUB obtained to evaluate response to therapy. KUB demonstrates bilateral residual stone fragments
  - Evaluate the structure of the spine
  - Evaluate retroperitoneal air and fat-fascial planes (abscess, infection, perforation)
  - Detect air in the GU tract or free air in the abdomen
  - Impacted feces (constipation)
  - Calcifications (urolithiasis)
  - Detect retained foreign bodies
  - Evaluate the position of stents or drains
- Plain film X-ray of the pelvis
  - To evaluate the bony pelvis and the terminal spine
  - Evaluate for any calcifications or foreign body within/adjacent to the urinary bladder or urethra

The primary indications for plain radiographic imaging in pediatric urology are:

- Two-positional abdominal plain film or KUB (Kidney-Ureter-Bladder) [Figure 1] to visualize any radio-opaque objects in the GU tract or in the abdomen.

## CONTRAST STUDIES

Diagnostic imaging modalities that employ contrast agents, permit visualization of the structural details of internal organs that would not otherwise be demonstrable. There have been several advances in this aspect of diagnostic imaging, including improved contrast agents and improvements in the fluoroscopy equipment used in this modality (low dosimetry, digital equipment and rotational fluoroscopy). Modern contrast agents contain as much as 2,000 times the iodine as the total physiological body content, but these agents are rapidly and safely cleared from the body without any adverse effects to the patient, in most instances. Contrast agents are categorized according to their chemical structure and relative osmolality, into high and low osmolality agents. These contrast agents are further subdivided into ionic and nonionic agents. The high-osmolality agents are associated with a higher risk of adverse events (nephrotoxicity, idiosyncratic reactions), while the low-osmolality are better tolerated with less discomfort and fewer cardiovascular and anaphylactic-type reactions, however, they are much more expensive.[4]

The common studies employed in pediatric urology that require the administration of contrast agents include:

- Intravenous urography (IVU)
- Voiding cystography (VCUG - Voiding cystourethrogram, MCUG - Micturating cystourethrogram)
- Retrograde urethrogram (RUG)
- Cystogram
- Whitaker test
- Angiography
- Computed tomography
- Interventional radiological studies (Antegrade pyelography)

## INTRAVENOUS UROGRAPHY OR INTRAVENOUS PYELOGRAPHY

Intravenous urography (IVU) (aka IVP) or excretory urography is one of the oldest uroradiologic imaging modalities. Until the advent of MR urography and CT urography, this was the only means by which to evaluate both the morphology and the function of the upper urinary tract. However, due to concerns of radiation exposure in children and the advent of the newer modalities, the indications for IVU have evolved and it should be tailored to answer the individual patient's clinical query.[5,6]

The current indications for IVU in a pediatric patient are:

- Hematuria associated with renal colic (although a non-contrast CT scan [stone protocol] is deemed to have higher diagnostic accuracy in these cases).
- Symptomatalogy suggesting specific congenital anomalies (e.g. constant dribbling of urine in a girl - ? ectopic ureter).
- Abnormal findings on a renal ultrasound that warrant further evaluation (e.g. calyceal diverticula).
- History of papillary necrosis, tuberculosis of the GU tract, medullary sponge kidney.

## VOIDING CYSTOURETHROGRAPHY

Voiding cystourethrography (VCUG or MCUG) remains the mainstay of uroradiology in children and should be performed after a thorough sonographic evaluation of the kidneys and bladder. The ‘ALARA’ principle for reducing radiation exposure has led to the use of digital fluoroscopes optimized for use in children that are capable of pulsed fluoroscopy but maintain diagnostic image quality.[7,8] The primary indications for performing a VCUG are a history of a febrile urinary tract infection (UTI) or prenatally detected hydronephrosis. The VCUG is the only diagnostic imaging modality that can reliably directly demonstrate posterior urethral valves. An adequately performed VCUG provides anatomic and functional information about the urinary bladder (bladder capacity, vesico-ureteral reflux (VUR), trabeculations, diverticula or ureteroceles) [Figure 2d] and the urethra (valves, strictures, incomplete relaxation of the sphincter or prostatic utricle).

**Figure 2a:**
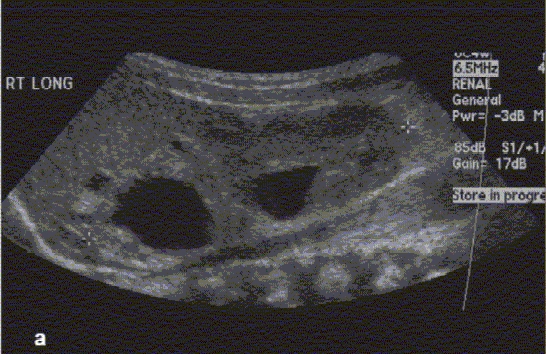
Renal-Bladder Ultrasound and VCUG. One-month-old female child with prenatally detected hydronephrosis. Renal and bladder ultrasound (Figures 2a–c) demonstrate bilateral renal duplication with upper poles subtended by a ureterocele. VCUG (Figure 2d–e) demonstrates bilateral ureteroceles with left lower pole VUR (Figure 2a) Renal Ultrasound demonstrating right renal duplication

**Figure 2b:**
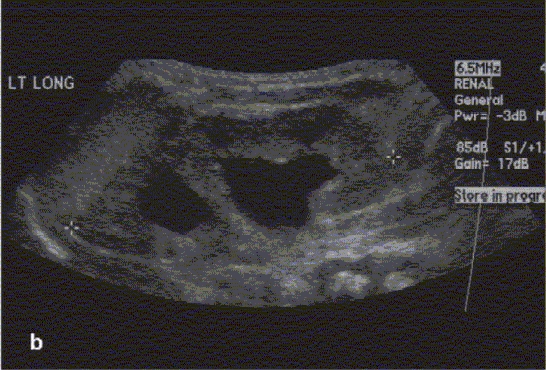
Renal ultrasound demonstrating left renal duplication

**Figure 2c:**
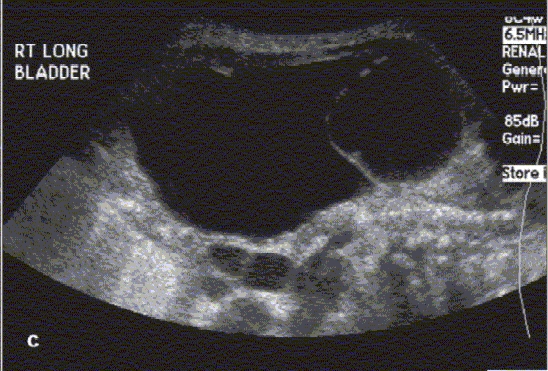
Bladder ultrasound with ureterocele arising from right side of bladder

**Figure 2:**
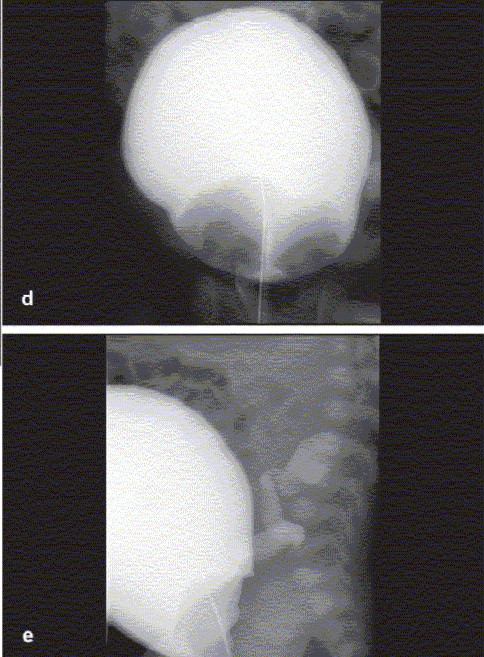
(d) VCUG demonstrating bilateral ureteroceles and (e) left lower pole vesicoureteral reflux

**Figure 3:**
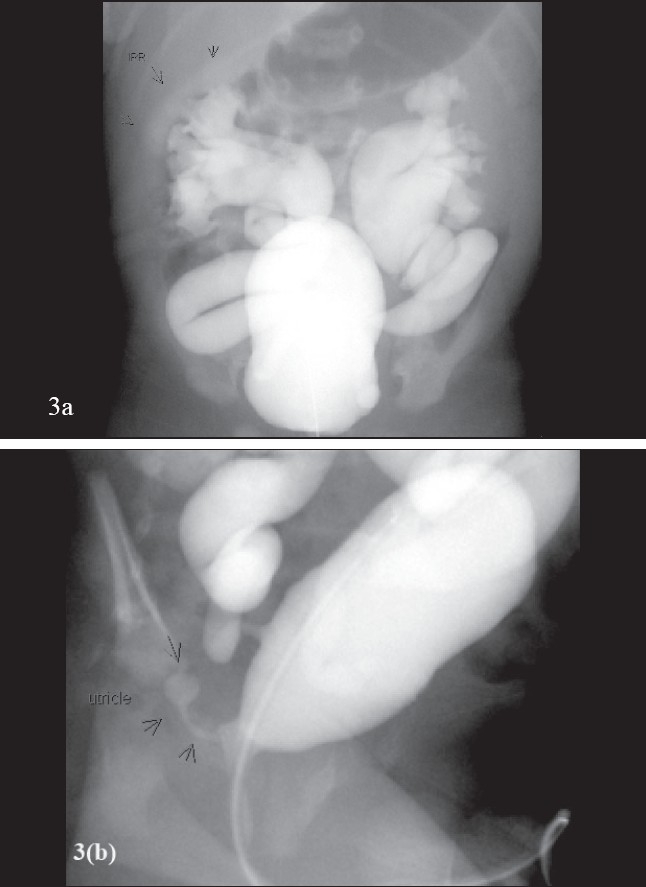
VCUG. Male infant with prenatally detected bilateral hydronephrosis. VCUG demonstrates bilateral Grade 5/5 VUR. Additional information includes intra-renal reflux in the right kidney and also a prominent utricle with reflux of contrast into it. (a) VCUG with bilateral Grade 5/5 VUR and right intra-renal reflux. (b) VCUG demonstrating reflux into the utricle

The selection of contrast media is critical to the quality of the study obtained; the medium must be radiopaque, sufficiently viscous to demonstrate bladder and urethral anatomy. It must also be well tolerated by the urothelium and harmless in case it does gain intravascular access. The VCUG in addition to detecting VUR, also aids in the grading and classification of the VUR [Figure 2d and 2e].[9] Additionally, in the presence of VUR, the VCUG can assess for the presence of intrarenal reflux (reflux of contrast into the collecting ducts or ducts of Bellini, this finding automatically raises the grade of VUR to grade 5/5) [Figure 3a] and for drainage of the refluxed material to exclude concomitant uretero-vesical junction (UVJ) or uretero-pelvic junction (UPJ) obstruction, especially in the high grades of VUR.

The scout film of the VCUG also aids in the diagnosis of constipation, that should raise the suspicion of dysfunctional elimination syndrome or any bony abnormalities that might suggest spinal cord anomalies (tethered spinal cord or occult myelomeningocele.[10] The post-void films of the VCUG are useful in demonstrating ‘vaginal voiding’, incomplete emptying of the urinary bladder suggesting some anatomic or functional obstruction of the bladder neck or urethra (e.g. detrusor sphincteric dysfunction) and also reflux of contrast into the prostatic or ejaculatory ducts suggesting high-pressure voiding [Figure 3b]. It has been shown that there is a diagnostic advantage to infusing warmed contrast medium versus room temperature contrast medium.[11]

Frequently, children referred for VCUG are already on treatment with antibiotics (either treatment or prophylactic doses), however, children with a prosthetic heart valve, a valvular lesion, a septal defect or patent ductus should receive prophylactic antibiotics to prevent endocarditis.

In order to increase the diagnostic yield of detecting VUR, the radiologist should, in children with a high degree of clinical suspicion of having VUR, perform a cyclical VCUG. This involves multiple cycles of bladder filling and emptying. However, one must keep in mind that each cycle incrementally increases the radiation exposure of the child.[12,13] Typically the VCUG is obtained several weeks after a febrile UTI, however it can also be safely performed during an active UTI under the protection of intravenous antibiotics. In fact there is a subset of patients that only develop secondary VUR when actively infected.

## RETROGRADE URETHROGRAM AND CYSTOGRAM

These diagnostic studies are mainly indicated in the evaluation of GU trauma, however due to the rapid acquisition of images by a spiral CT scan, the utility of these studies has been significantly reduced. Most cystograms in trauma patients are now CT cystograms. To ensure an adequate study in an adult patient a minimum of 300 ml of contrast should be instilled in the bladder, for the pediatric patient the bladder should be filled to the age appropriate expected bladder capacity [(age in years + 2) × 30 mL].[14]

Retrograde urethrograms (RUGs) are also useful in defining the extent and nature of urethral stricture disease in conjunction with a cystogram. In cases of patients with a history of multiple urethroplasties for a hypospadiac urethra, an RUG can delineate the existing urethral anatomy prior to reconstructive surgery. We usually perform elective cystograms utilizing the three-dimensional rotational fluoroscopic technique (described in detail later in this article). This technique offers a significant enhancement of the anatomic detail obtained from the cystogram and aids in the diagnosis and preoperative surgical plan.

## WHITAKER TEST (ANTEGRADE PERFUSION PRESSURE FLOW TEST)

The Whitaker test is an invasive test performed to evaluate the dynamics of either a UPJ obstruction or a UVJ obstruction. This study involves percutaneous access into the pelvicalyceal system. Contrast medium is instilled into the collecting system while monitoring the intrarenal and intravesical pressure. The opening pressure of the renal pelvis or the ureter is evaluated and can aid in the diagnosis of obstruction at these sites. The Whitaker test has limited applicability in the pediatric age group (due to the development of the noninvasive diuretic renogram), however it can be useful in instances of recurrent UPJ obstruction after a pyeloplasty or when the diuretic renogram is non-diagnostic in a symptomatic patient in whom a UPJ obstruction is clinically suspected.[15,16]

## ANGIOGRAPHY

Due to the invasive nature of this imaging modality it has very limited application in pediatric uroradiology. The main indications include:

- Diagnosis of arterio-venous malformations in instances of gross hematuria (post-traumatic, congenital or after renal biopsy)
- It can be both diagnostic and therapeutic (permitting selective embolization of injured vessels) in certain instances of renal trauma with segmental artery bleeding
- Evaluation of reno-vascular hypertension
- Evaluation of pseudo-aneurysm in renal transplants

## ULTRASOUND

Medical ultrasonography (US) was introduced after the Second World War and is now the imaging modality of choice in pediatric uroradiology due to its noninvasive nature. It is mainly used as the primary screening diagnostic modality that then dictates further imaging strategies. Due to its noninvasive and non-ionizing nature it can be used for repeated follow-up imaging of the GU tract. The current generation of US equipment has benefited immensely from the advances in microchip technology and electronic miniaturization, allowing unprecedented image quality at greater tissue depths and resolution.

Indications for US evaluations include:

- Prenatal imaging (the GU tract can be imaged as early as 14–16 weeks post-conception, with renal parenchymal differentiation seen by 20 weeks of gestation)[17]
- Screening of the GU tract for congenital anomalies in neonates with a history of an abnormal prenatal US (or a sibling with VUR)
- Anatomic survey of the retro-peritoneum in a child presenting with an abdominal mass (evaluate the kidney and adrenal gland for tumors or cystic masses)
- Evaluation of a febrile UTI (color Doppler imaging to detect pyelonephritis or hematuria[18]
- Evaluation of renal colic (presence of ureteral jets suggests insignificant obstruction if any)
- Follow-up evaluation of known GU tract anomalies (e.g. renal cyst, renal duplication, renal dysplasia, hydronephrosis, hydroureter, ureterocele, urachal remnants) [Figure 2abc, & Figure 5ab]

**Figure 4:**
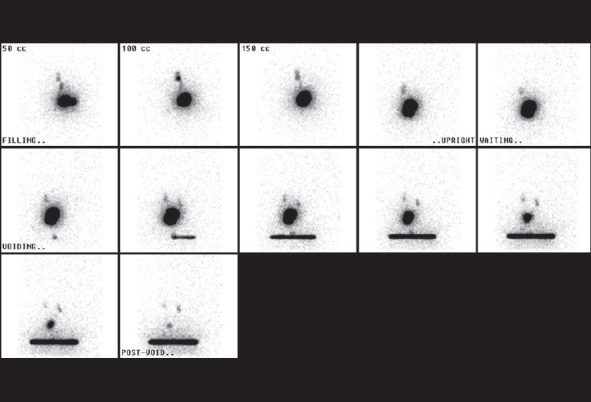
Radionuclide cystogram. Radionuclide cystogram (RNC) demonstrating moderate grade bilateral VUR (Grade 2–3/5)

**Figure 5:**
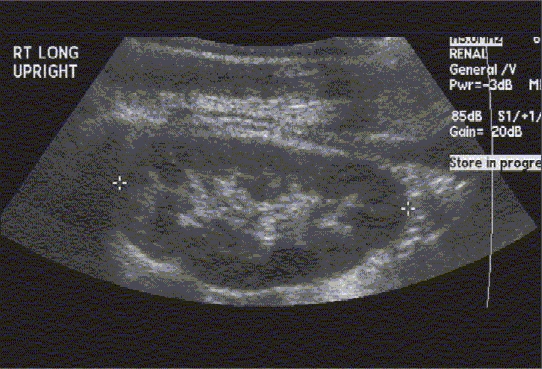
Renal Ultrasound, Lasix Renogram and Retrograde Pyelogram Patient is a 14-month-old infant born with prenatally detected hydronephrosis. Postnatal evaluation demonstrated mild left hydronephrosis. Serial US demonstrated progressive left hydronephrosis (b). Lasix Renogram demonstrated a significant increase in the T1/2 drainage of the left renal pelvis (102 min) (c). A left retrograde pyelogram demonstrates obstruction of the left UPJ (d). (a) US demonstrating normal right kidney

**Figure 5b:**
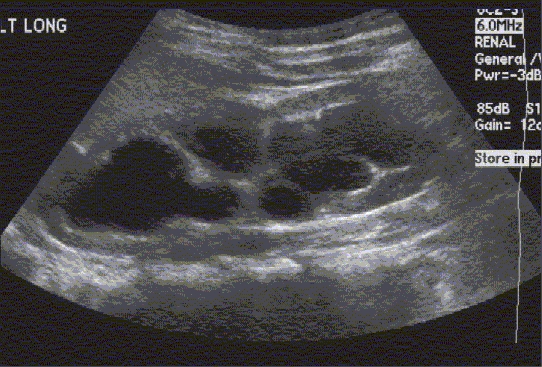
US demonstrating left hydronephrosis (Grade 3 of 4 society of fetal urology grading system)

**Figures 5(c-d):**
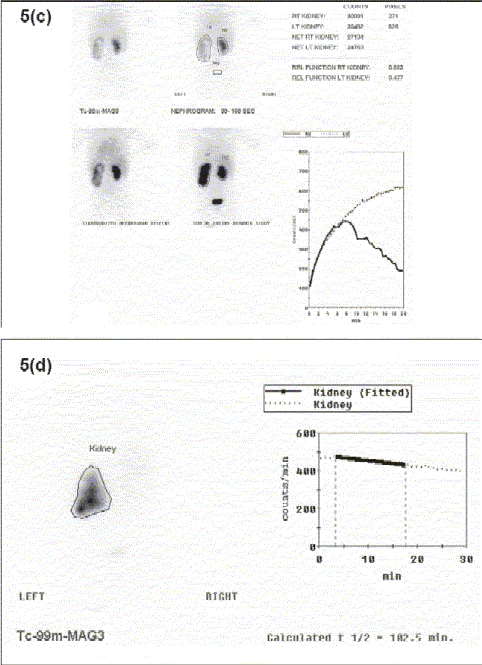
Lasix Renogram demonstrating left kidney with prolonged T1/2 drainage

**Figure 5e:**
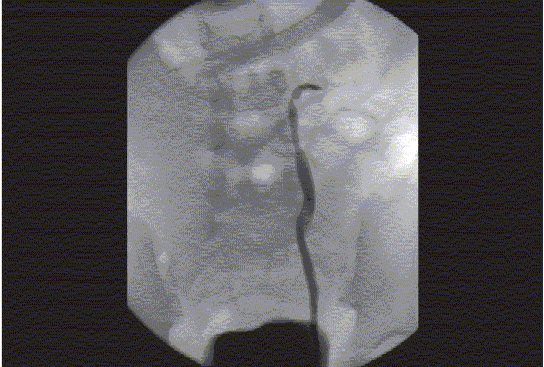
Retrograde pyelogram demonstrating high-grade obstruction at the level of the left UPJ. The retained urine immediately dilutes the contrast that enters into the dilated left renal pelvis

**Figure 6a:**
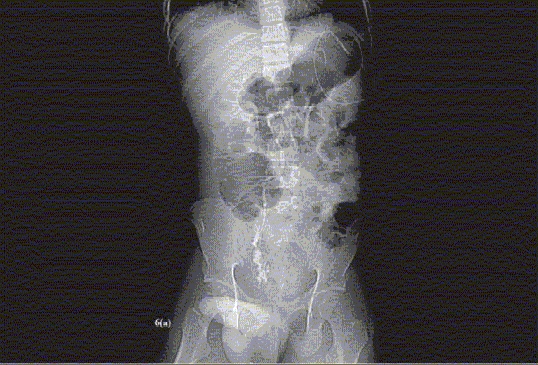
KUB, CT scan, Retrograde Pyelogram, Antegrade Nephrostogram and Rotational Fluoroscopic Tomography. The patient is a teenage male who had sustained penetrating trauma to his perineum. A tree branch penetrated through his perineum, into the right lower quadrant of the abdomen, avulsing his right ureter from the bladder at the UVJ. (a) KUB obtained after IV contrast administered for CT scan. Image demonstrates extravasation of contrast from the distal right ureter

**Figure 6(b-c):**
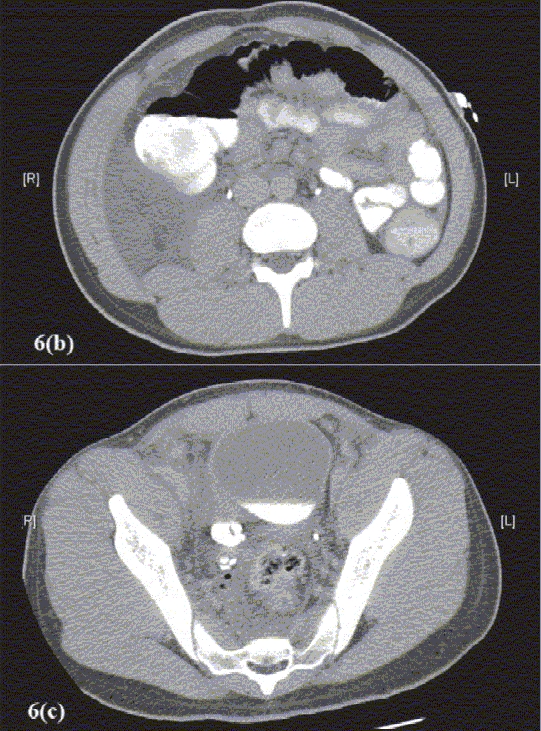
Axial sections of CT scan demonstrating normal ureteral anatomy in the abdomen (b), but the pelvic portion of the right ureter has contrast extravasating at the level of the right UVJ (c)

**Figure 6d:**
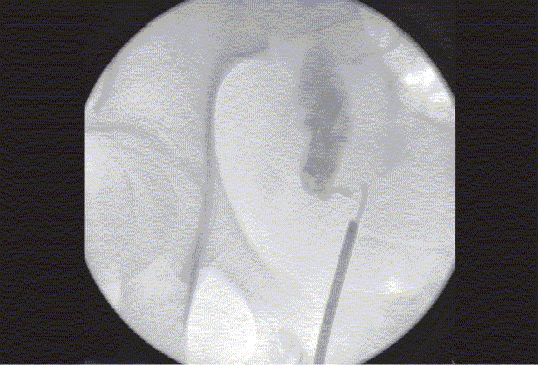
Retrograde pyelogram demonstrates complete separation of the distal right ureter from the UVJ

**Figure 6e:**
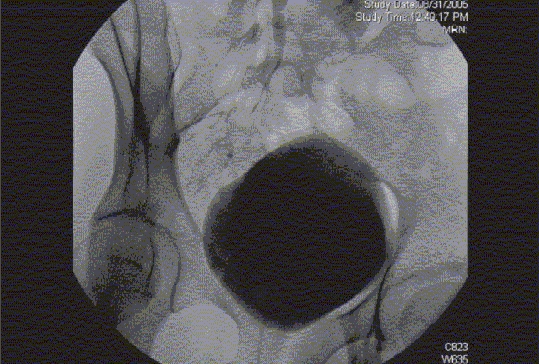
Antegrade contrast study performed via right nephrostomy tube, demonstrating the atretic narrow lumen of the distal right ureter. This study was performed in order to aid the preoperative planning

**Figure 6f:**
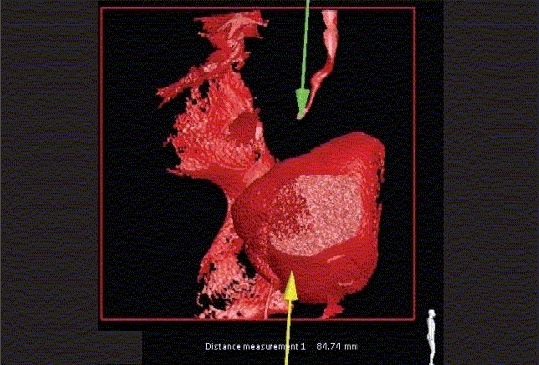
Three-dimensional rotational fluoroscopic tomography image demonstrating the anatomy of the distal right ureter in relationship to the bladder. This study demonstrates the actual distance to be bridged surgically during the reconstructive procedure

**Figure 7:**
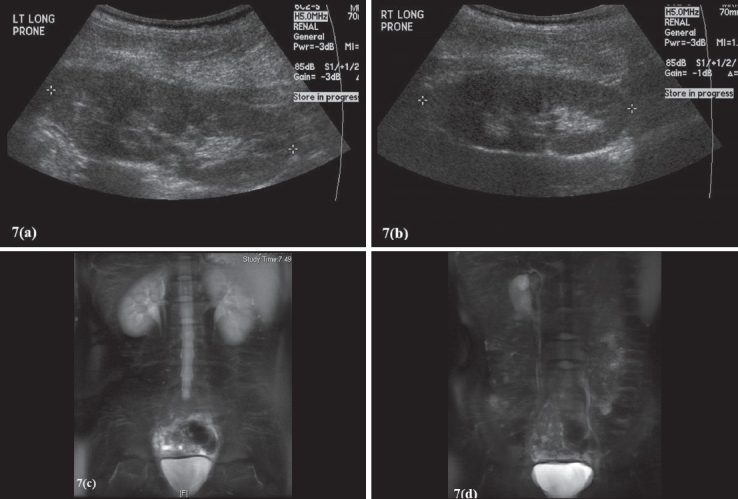
Renal US and MR Urogram. Patient is a nine-year-old female who presented with constant dribbling of urine. Her US demonstrated normal upper tracts(a-b). MR urogram demonstrates right renal duplication with an ectopic right upper pole ureter (c-d). (a-b) Renal US demonstrates normal kidneys, no evidence of duplication. Figs 7(c–d) MR urogram (T2 weighted imaging sequence) demonstrating duplication of the right kidney (c), with an ectopic insertion of the right upper pole ureter into the bladder neck (d)

**Figure 8:**
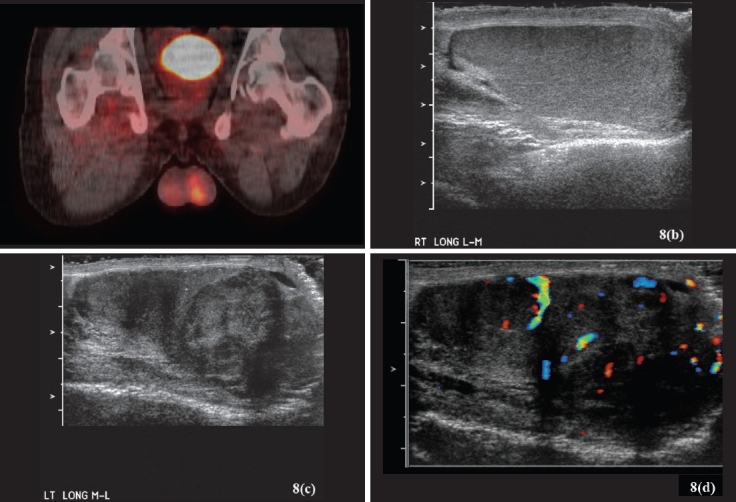
PET scan and scrotal US. Patient is a 19-year-old male with cerebral palsy and a history of neurofibromatosis. He underwent a PET scan to evaluate a retroperitoneal mass. The PET scan(a) demonstrated increased uptake by the left testis (the increased area of signal in the pelvis is the urinary bladder that lights up as the tracer is excreted). Physical examination and a scrotal US (b-d) demonstrated a left testicular mass. The patient underwent a left radical orchidectomy and was found to a have Stage 1 seminoma. (a) A maximum intensity anterior projection image of a PET scan with 2 deoxy-2-fluoro-D-Glucose (FDG) demonstrating area of increased activity in the left testis. (b-d) Scrotal US images demonstrating normal homogenous appearance of the right testis(b). The left testis demonstrates loss of homogenicity and a discrete mass with normal blood flow pattern in the lower pole region(c-d)

**Figure 9:**
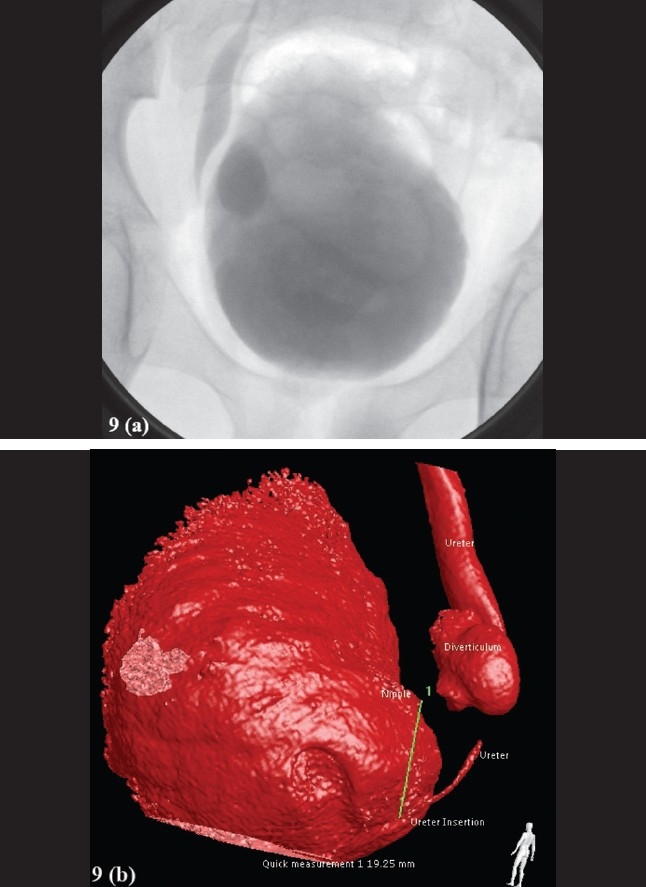
Cystogram and Rotational Fluoroscopic Tomography. Patient presented with recurrent UTI. Initial evaluation included a renal US that was normal. His VCUG demonstrated a sizable diverticulum (that increased in size after voiding) on the right side of the bladder adjacent to the UVJ(a). No VUR was identified. In order to plan the surgical approach and evaluate the need for ureteral reimplantation, a RFT study (b) was performed, the results permitted an extravesical repair. (a) Cystogram in combination with IV administration of contrast (IVP) demonstrating the diverticulum and the right ureter. (b) RFT scan demonstrating that the rightsided bladder diverticulum is not a Hutch diverticulum
- Evaluation of blood flow patterns of the kidney with color Doppler (to detect pyelonephritis, renal vein thrombosis and to ensure renal transplant viability - calculate the resistive index)
- Post void residual evaluation in patients with dysfunctional voiding
- Evaluation of the internal genitalia (in cases of intersex/ambiguous genitalia); in female neonates, the endocrinological stimulation by the maternal hormones causes the uterus to be visualized as a bulbous enlarged organ with a prominent endometrial stripe
- Evaluation of testes (structure and blood flow patterns - torsion, tumors) [Figure 8b, c, d
- Evaluation of urethral disease (transperineal US to evaluate for PUV or urethral stricture disease)[19]
- Evaluation of constipation[20]
- Evaluation of the spinal cord in cases of suspected tethered cord (up to six months - after that the vertebral bodies are calcified and an MRI is required)[21]
- Intraoperative localization of soft tissue lesions (testicular, adrenal or renal lesions)

The differential diagnosis of a renal mass in the neonatal patient, that warrants an evaluation with ultrasonography includes:

- Urinary tract obstruction
- Uretero-pelvic junction (UPJ) obstruction
- Uretero-vesical junction (UVJ)obstruction
- Posterior Urethral valves (PUV)
- Urogenital sinus
- Cloacal anomalies
- Reflux with hydronephrosis
- Multicystic dysplastic kidney (MCDK)
- Congenital mesoblastic nephroma
- Nonpolycystic nephromegaly
- Neuroblastic nephroma
- Renal cystic disease

There has been a significant push to obviate the need for invasive ionizing radiographic studies (VCUG or nuclear cystograms) for the detection of VUR by using contrast enhanced US (cystosonography with echocontrast).[22,23] While there have been many studies that have demonstrated a proof of concept, this modality has not yet attained widespread clinical applicability.

## COMPUTERIZED TOMOGRAPHY SCANS

Computerized tomography (CT) has limited application in the evaluation of the GU tract of neonates due to the lack of significant amounts of intraperitoneal and retroperitoneal fat; this necessitates the administration of contrast to visualize solid organs and vessels. However, CT imaging has become vital in the diagnostic evaluation of urolithiasis (non-contrast CT scan - stone protocol) and in cases of abdominal trauma [Figure 6b and c]. The urinary tract is the second most commonly injured organ system in children (the central nervous system is the most commonly injured organ in blunt trauma). Up to 5% of all trauma-related fatalities in the pediatric age group in the US are secondary to significant GU trauma. Preexisting GU abnormalities predispose children to renal injuries from blunt trauma, these include hydronephrosis, tumors or compensatory hypertrophy.

Computerized tomographic imaging is a procedure that involves a high radiation dose.

Table 1 demonstrates that one abdominal CT scan involves an effective radiation dose equal to 500 chest radiographs and is the equivalent of the average national (in the US) background environmental radiation received over a period of more than three years.

**Table 1:** Computerized tomography (CT) radiation dosing (contrasts effective doses for chest X-rays with an abdominal CT scan)[24,25]

Radiographic procedure	Effective dose in sieverts (Sv)*	Chest X-ray equivalent	Equivalent background radiation time
Chest X-ray (P-A view)	0.02	1	2.4 days
Chest CT scan	8	400	2.7 years
Abdomen CT scan	10	500	3.3 years

* 1 sievert (Sv) = 1 gray (Gy) of X-rays (it is a dose equivalent rather than the actual physical absorbed radiation)

Effectively, a single abdominal CT scan has the equivalent radiation exposure as 500 chest X-rays.[25] The importance of reducing the radiation dose in pediatric patients has already been discussed. As a rule of thumb, the lifetime cancer mortality risk attributable to the radiation exposure from a single abdominal CT examination in a one-year-old child is of the order of one in a 1000; it can be significantly reduced if every effort is made by the radiologist to reduce the radiation dose by adjusting the mAs and the kVp to the size of the patient.[26] The advent of helical CT scanners has allowed for cross-sectional imaging in children, with a very short exposure time that decreases the overall radiation exposure to the patient and obviates the need for sedation. The latest generation of multidetector CT scanners (MDCT) allow for rapid and complete imaging of the abdominal cavity with accurate definition of the retroperitoneum. When contrast is required oral contrast should be ideally given two to five hours prior to scanning while intravenous contrast is administered during the actual image acquisition. The MDCT scanners acquire tomograms in thicker slices than prior generations of scanners, thereby exposing the patient to less radiation per scan and permitting the reconstruction of thin slices with fine detail.

Computerized tomography imaging without contrast allows the visualization of calcifications within the GU tract (urolithiasis, nephrocalcinosis and metastatic calcifications), whereas contrast enhanced CT imaging enables the demonstration of infections or masses within the GU tract.

Multidetector CT has in most instances replaced IVP as the contrast imaging of choice for the GU tract. When interpreting the pediatric CT scan one should bear in mind that most children have significantly less retroperitoneal fat than adults, this does alter some of findings that are considered pathognomonic for certain conditions (e.g. retroperitoneal streaking in the peri-renal or peri-ureteral region is considered a good indicator of a high-grade obstruction in adults, this is not always evident in children even with complete obstruction). The visualization of the intraperitoneal organs is an additional benefit of the CT scan especially in the evaluation of acute flank pain, as it demonstrates the appendix and the adnexal organs (appendicitis, pelvic inflammatory disease).[27,28] Sagittal, coronal or three-dimensional reformatting of the two-dimensional tomographic images of a CT scan provides enhanced diagnostic utility of this modality.

## NUCLEAR MEDICINE IMAGING

Diagnostic imaging with radionuclide tracers offers functional information that is not attainable with traditional radiographic imaging, in addition to reduced radiation exposure. Nuclear imaging is usually reserved for the older pediatric patient as it has very limited utility in the neonatal period (due to the inability of these tests to provide adequate anatomical detail). The most common nuclear imaging studies include radionuclide cystography (RNC), cortical renal scintigraphy and diuretic renography (Lasix Renogram).

Radionuclide cystography (RNC) for diagnosing VUR[29] is indicated in instances of:

- Family screening for vesicoureteral reflux
- Follow-up of known VUR [Figure 4]
- Follow-up of patients after anti-reflux surgery (e.g. ureteral reimplantation or Deflux™ injection)

The benefits of RNC over VCUG are:

- It has a significantly lower radiation dose
- It provides continuous monitoring of bladder filling and voiding

The RNC does not provide the same anatomic details that the VCUG is capable of demonstrating, e.g., presence of a para-ureteral diverticulum, bladder trabeculation, spinal anomalies, etc.

Renal cortical scanning is used to identify anomalies of the upper urinary tract that affect renal function e.g. multicystic dysplasia and pyelonephritic scarring. Tubular transport tracers include Technetium [^99m^Tc], Mercaptoacetyltriglycine [MAG-3] and ^99m^Tc Dimercaptosuccinic acid [DMSA]; these agents identify renal cortical tissue and can localize ectopic renal tissue. The DMSA scan is the most accurate imaging modality for the diagnosis of acute pyelonephritis, the decreased accumulation of the tracer in the renal parenchyma is secondary to inflammatory edema, the resultant deceased blood flow and cellular enzymatic activity.[30] When evaluating a neonate, it is preferable to use the cortical agents as these patients usually have a low glomerular filtration rate.

Diuretic Renography requires the use of glomerular filtration tracers (^99m^Tc Diethylenetriaminepenta-acetic acid [DTPA]) and is mainly used to evaluate hydronephrosis as it can distinguish obstructive conditions from physiological hydronephrosis (secondary to either ureteropelvic junction obstruction or urterovesical junction obstruction). The ‘Well Tempered Renogram’ is the result of standardization of this study by the Pediatric Imaging Council of the Society of Nuclear Medicine.[31,32] The procedure protocol calls for very specific hydration parameters and the presence of an indwelling catheter in the bladder. These studies provide information about the differential function of the kidneys and also the drainage time of the collecting system (t 1/2) [Figure 5c] and d].

## MAGNETIC RESONANCE IMAGING

Magnetic resonance (MR) imaging of the abdomen in the hands of an experienced radiologist can provide images of the GU tract that have exquisite detail. The limitations of MR imaging are:

- Motion artifact (vascular pulsations, peristalsis, respiration motion)
- Need for sedation or general anesthesia in children less than seven years of age
- Expensive equipment and infrastructure (special rooms that don't interfere with the magnetic field of the human body, computer archiving systems to store the imaging data) often limit the accessibility to this imaging modality.

**Figure 10:**
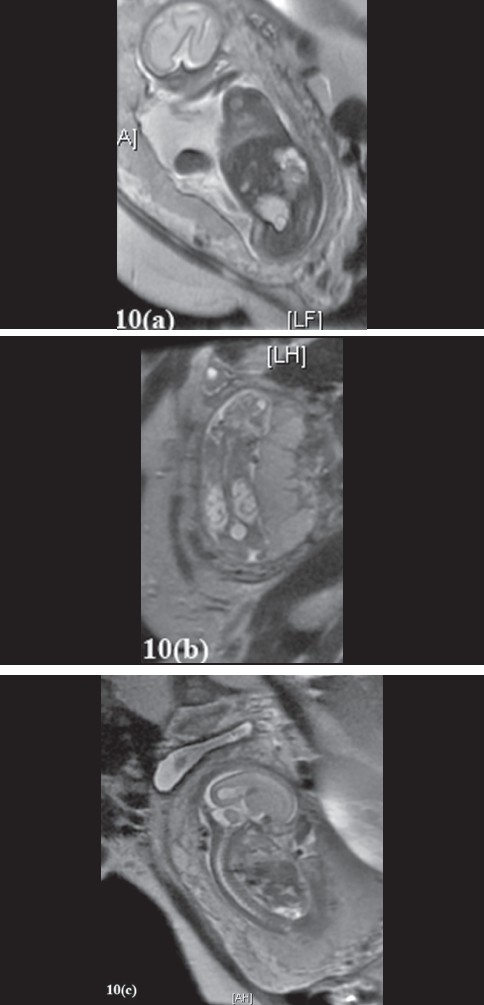
Fetal MR scans (FMRI) to detect prenatal pathology FMRI scans are rapidly gaining popularity as the definitive prenatal imaging modality. (a-c) demonstrate a three cases of in-utero GU tract pathology that we have diagnosed over the past few years with FMRI. (a) FMRI demonstrating a singleton fetus with a duplicated left kidney with a ureterocele seen in the bladder and a normal posterior urethra is visualized. The amniotic fluid index is at the low end of normal. (b) FMRI demonstrating a singleton male fetus with a distended bladder and bilateral hydroureteronephrosis. There is also significant oligohyradamnios, all these findings should raise the suspicion of a bladder outlet obstruction secondary to posterior urethral valves. Fetal urinary values should be evaluated prior to any in-utero interventions (vesico-amniotic shunting or fetoscopic valve ablation). (c) FMRI demonstrating a singleton fetus with severe oligohyradamnios (gender not able to be determined due to fetal position and low fluid), an empty bladder and bilateral small cystic kidneys are seen in addition to low lung volumes, all these findings should raise the suspicion of significant renal dysplasia. There are no interventions for this scenario and it is associated with a 100% fetal mortality rate

Magnetic resonance imaging is the modality of choice for pelvic imaging (internal genitalia, ectopic ureters) [Figure 7]. Magnetic resonance urography is a rapidly expanding field of study; due to the absence of radiation exposure to the patient, it is being used to evaluate the presence of ureteropelvic junction obstruction.[33] Imaging of fetal anatomy in-utero while possible with US, is significantly enhanced with fetal MR imaging (FMRI). This is attributable to the fact that the conditions that hinder US imaging i.e. a lack of fluid and patient obesity are almost always present when dealing with in-utero GU pathology. Fetal MR imaging is still considered to be an experimental imaging protocol. At our institution we perform FMRI after the 18th week of gestation and utilize MRI sequences such as ‘Single Shot Fast Spin Echo (SSFSE)’ with T2 imaging. The image acquisition is very fast, permitting these studies to be performed without the need for maternal sedation. In a review of our institutional series of over 100 studies, FMRI had a 100% accuracy rate compared to a 55% accuracy rate for prenatal US.[34,35] Fetal MR imaging should be considered as a complementary study for prenatal US and not a replacement [Figure 10].

## ROTATIONAL FLUOROSCOPIC TOMOGRAPHY

Rotational fluoroscopic tomography (RFT) involves continuous digital image acquisition as a fluoroscopic c-arm rotates around a patient during either intravascular or intraluminal contrast injection. Subsequent three-dimensional (3D) reconstructions can be performed within four minutes of image acquisition. Rotational fluoroscopic tomography technology is a powerful real time tool with diagnostic and therapeutic applications. It provides enhanced spatial orientation, improved diagnostic capability and assists in interventional radiology or surgical planning. At our institution we perform RFT and 3D reconstructions in a Philips Integris Allura™ interventional suite. Studies that can be performed with RFT include antegrade pyelography, evaluation of UPJ or UVJ obstruction.[36,37] Non-urological applications include percutaneous transhepatic cholangiography, evaluation of recto-urethral fistulae, anal atresia and comminuted fractures. The radiation exposure of an RFT study is one-sixth that of a CT scan [Figure 6f and 9b].

## POSITRON EMITTED TOMOGRAPHY SCAN

Positron emitted tomography (PET) scan is a diagnostic examination that involves the acquisition of physiologic images based on the detection of radiation from the emission of positrons (tiny particles emitted from a radioactive substance, usually a glucose analog 2-deoxy-2-[18F] fluoro-D-glucose (FDG) that is administered to the patient). Positron emitted tomography scans permit the study of organ function by detecting alterations in biochemical processes that suggest disease before changes in anatomy are apparent with other imaging tests, such as CT or MRI. The PET scans are used most often to detect cancer and to examine the effects of cancer therapy by characterizing biochemical changes in the cancer [Figure 8].[38] Proper interpretation of FDG PET images requires knowledge of the normal physiologic distribution of the tracer, frequently encountered physiologic variants and benign pathologic causes of FDG uptake that can be confused with a malignant neoplasm.[39] Different colors or degrees of brightness on a PET image represent different levels of tissue or organ function. For example, because healthy tissue uses glucose for energy, it accumulates some of the tagged glucose, which will show up on the PET images. However, cancerous tissue, which uses more glucose than normal tissue, will accumulate more of the substance and appear brighter than normal tissue on the PET images. The PET scans are mainly utilized in the evaluation of retroperitoneal tumors and renal tumors in the pediatric GU patient. Ideally the PET scan is utilized as part of a larger diagnostic work-up, which permits comparison of the PET scan with other imaging studies, such as CT or MRI, enhancing the diagnostic accuracy of the PET scan.

In summary pediatric imaging continues to evolve as technological innovations are incorporated into clinical imaging modalities. A thorough understanding of the contemporary imaging techniques, their indications and limitations will enable the surgeon to tailor the appropriate combination of diagnostic tests for any given patient, bearing in mind the potential problems and costs associated with these imaging modalities. When treating pediatric patients, it is the responsibility of all healthcare professionals to ensure that the ‘ALARA’ principle is followed whenever radiographic studies are obtained.
